# Supplementary material for: Inhibition of DUSP6 Activates Autophagy and Rescues the Retinal Pigment Epithelium in Sodium Iodate-Induced Retinal Degeneration Models In Vivo and In Vitro
Source: Biomedicines. 2022 Jan 12;10(1):159. doi: 10.3390/biomedicines10010159 (PMC8773272; doi:10.3390/biomedicines10010159)
Supplement: Supplementary file 1 [file biomedicines-10-00159-s001.zip › biomedicines-1519184-supplementary.pdf]

Supplementary Materials

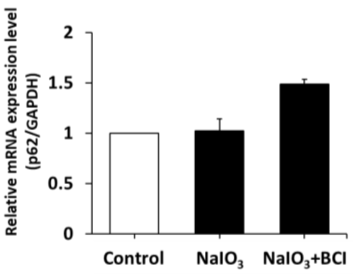

Supplementry Figure S1. BCI upregulated the p62 mRNA level under oxidative stress. ARPE-19 cells were co-treated with BCI and $NaIO_3$ for 6 hours and the mRNA levels of p62 were detected by qPCR.

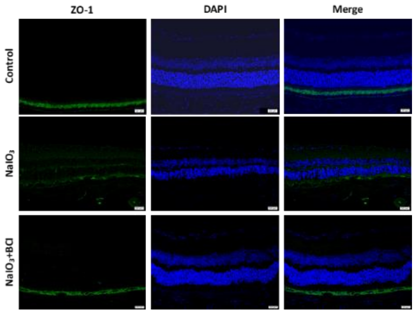

Supplementary Figure S2. Cell-cell junction were damaged after treated with $NaIO_3$ and co-treated with BCI rescue the cell-cell junction between RPE cells. Scale bar = 20 μm.

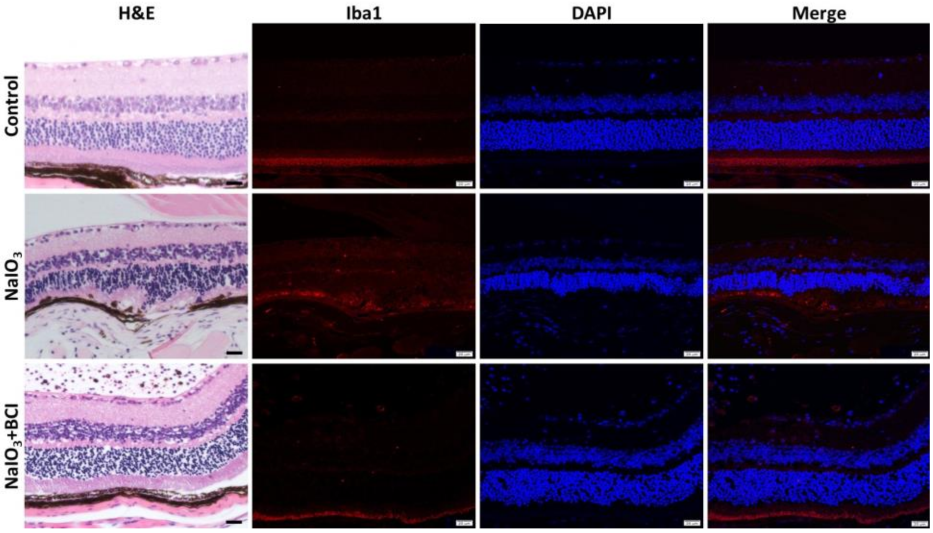

Supplementary Figure S3. Immunohistochemical examinations of the expression of Iba1 in $NaIO_3$-treated mice retina. Representative immunohistochemical staining for Iba-1 (red) and DAPI (blue) 7 days after 30 mg/kg $NaIO_3$ injection. Iba-1 were used for microglial markers. Scale bar = 20 μm.
